# Bone metastases are associated with worse prognosis in patients affected by metastatic colorectal cancer treated with doublet or triplet chemotherapy plus bevacizumab: a subanalysis of the TRIBE and TRIBE2 trials

**DOI:** 10.1016/j.esmoop.2022.100606

**Published:** 2022-10-31

**Authors:** E. Dell’Aquila, D. Rossini, C.A.M. Fulgenzi, A. Passardi, E. Tamburini, G. Vetere, M. Carullo, F. Citarella, C. Antoniotti, A. Zaniboni, F. Pietrantonio, A. Spagnoletti, F. Marmorino, B. Borelli, G. Allegrini, S. Lonardi, F. Nappo, G. Masi, C. Cremolini, D. Santini

**Affiliations:** 1 Medical Oncology 1, IRCCS Regina Elena National Cancer Institute, Rome, Italy; 2 Department of Medical Oncology, University Campus Bio-Medico of Rome, Rome, Italy; 3 Unit of Oncology, University Hospital of Pisa, Pisa, Italy; 4 Department of Translational Research and New Technologies in Medicine, University of Pisa, Pisa, Italy; 5 Department of Medical Oncology, IRCCS Istituto Romagnolo per lo Studio dei Tumori ‘Dino Amadori’, Meldola, Italy; 6 Department of Oncology and Palliative Care, Cardinale G. Panico Tricase City Hospital, Tricase, Italy; 7 Medical Oncology Unit, Fondazione Poliambulanza, Brescia, Italy; 8 Department of Medical Oncology, Fondazione IRCCS Istituto Nazionale dei Tumori, Milan, Italy; 9 Department of Oncology, Division of Medical Oncology, Azienda USL Toscana Nord Ovest, Livorno, Italy; 10 Oncology Unit 3, Department of Oncology, Veneto Institute of Oncology, IOV-IRCCS, Padua, Italy; 11 Oncology Unit 1, Department of Oncology, Veneto Institute of Oncology, IOV-IRCCS, Padua, Italy; 12 Department of Radiology, Oncology and Pathology, Policlinico Umberto, I Sapienza University of Rome, Rome, Italy

**Keywords:** colorectal cancer, bone metastases, prognosis, triplet chemotherapy

## Abstract

**Background:**

Colorectal cancer (CRC) is one of the most common cancers; ∼20% of patients have metastases at diagnosis, and 50%-60% subsequently develop metachronous metastases. Bone involvement, despite being rare, is usually associated with higher disease burden, worse prognosis, impaired quality of life, and significant health-related cost. In the last few years, following the positive results of the TRIBE and TRIBE2 trials, the association of FOLFOXIRI plus bevacizumab has become the new standard of care for metastatic CRC. Despite being highly efficacious in all subgroups, little is known about the activity of this regimen in patients with bone metastases.

**Patients and methods:**

We carried out a pooled analysis of TRIBE and TRIBE2 studies focusing on patients with skeletal deposits.

**Results:**

Our analyses on the whole population showed that patients with baseline bone involvement reported shorter overall survival [OS; 14.0 versus 26.2 months; hazard ratio (HR) 2.04, 95% confidence interval (CI) 1.46-2.87; *P* < 0.001] and progression-free survival (PFS; 6.2 versus 11.1 months; HR 1.96, 95% CI 1.42-2.69; *P* < 0.001) compared with those without bone metastases; no significant interaction with the treatment was reported for PFS (*P* = 0.094) and OS (*P* = 0.38). Bone metastases had a negative prognostic implication in the multivariate analysis (HR 2.24, 95% CI 1.54-3.26; *P* < 0.001). Furthermore, patients with bone lesions at first radiological progression (including those with baseline bone metastases) had a shorter OS compared with those who progressed in other sites (10.4 versus 13.2 months; HR 1.48, 95% CI 1.15-1.91; *P* = 0.002). A trend toward inferior OS (7.5 versus 11 months, HR 1.50, 95% CI 0.92-2.45; *P* = 0.10) appeared in patients with basal skeletal deposits compared with those with bone involvement at first radiological progression.

**Conclusions:**

Our study confirmed the negative prognostic impact of bone metastases in CRC. Furthermore, we demonstrated for the first time that the survival advantage of triplet chemotherapy plus bevacizumab is maintained even in this prognostically unfavorable subgroup.

## Highlights

• Bone metastases are associated with worse prognosis in patients affected by CRC.
• The prognostic impact of bone metastases is independent from baseline clinicopathological characteristics.
• The survival advantage of triplet chemotherapy plus bevacizumab is preserved even in patients with bone metastases.

## Introduction

Colorectal cancer (CRC) remains the third most common malignancy worldwide.^1^ About 15%-20% of patients are diagnosed with synchronous metastases^2^ and 50%-60% of patients develop metachronous metastases during disease course.^3^^,^^4^ Liver and lung are the primary sites of distant dissemination,^5^ whereas bone involvement is less common, in fact it is detected in only ∼10%-15% of patients, and is usually associated with localizations to other organs.^6^ According to a retrospective case series, 5.5% of patients have bone metastases at diagnosis.^7^ Despite not being common, skeletal involvement is associated with poor prognosis and impaired quality of life due to the risk of skeletal-related events (SREs), such as pain, spinal compression, or hypercalcemia, which often require hospitalization and cause high health-related cost.^8^ The incidence of SREs in CRC is not known; however, data from retrospective series report that ∼70% of patients with bone metastases experience SREs and radiation to the bone is the predominant one.^6^ In patients with metastatic CRC (mCRC) with bone metastasis, prognostic and predictive data from large clinical trials are currently lacking.^9^ Moreover, current data show that the incidence of bone metastasis in CRC has constantly increased in the last few years, possibly reflecting the improvement in overall survival (OS).^6^ These premises clearly highlight the urgent need to focus the attention on this growing group of patients.

In the past few years, FOLFOXIRI plus bevacizumab has become, according to international guidelines, one of the recommended first-line treatments for selected patients with mCRC.^10^^,^^11^ TRIBE and TRIBE2 were phase III randomized trials that demonstrated the superiority in terms of OS, progression-free survival (PFS), and objective response rate (ORR) of the triplet plus bevacizumab over the combination of doublet (FOLFIRI/FOLFOX) plus bevacizumab.^12^^,^^13^

The TRIBE trial^12^ reported an OS of 29.8 months in the triplet plus bevacizumab arm, compared with 25.8 months in the FOLFIRI plus bevacizumab group [hazard ratio (HR) 0.80, 95% confidence interval (CI) 0.65-0.98; *P* = 0.03]. In addition, PFS was as well improved to 12.1 months in the experimental arm, compared with 9.7 months in the control group (HR 0.75, 95% CI 0.62-0.90; *P* = 0.003). The advantage was confirmed in all the prespecified subgroups, independently from the molecular profile.^14^ The phase III TRIBE-2 trial showed that the frontline therapy with FOLFOXIRI plus bevacizumab followed by the same regimen reintroduction at first radiological progression was superior to the sequence of FOLFOX plus bevacizumab followed by FOLFIRI plus bevacizumab at progression of disease (PD).^15^ However, a better efficacy came at the expense of higher toxicities. The triplet therapy caused a higher incidence of neutropenia, diarrhea, and hypertension compared with the doublet group. In light of the aforesaid evidence, it is clear that there is a need to adequately stratify patients according to predictive and prognostic factors, sparing unnecessary toxicities to those who might not benefit from more intensive chemotherapy backbone.

It should be noticed that in the TRIBE and TRIBE2 trials no subgroup analysis was specifically carried out in patients with bone metastases, so there are no available data regarding the efficacy of triplet chemotherapy in this subset of patients.

Drawing from these considerations, we carried out a pooled analysis of the TRIBE and TRIBE2 trials to assess the prognostic and predictive relevance of bone metastases in patients with mCRC receiving upfront chemotherapy plus bevacizumab.

## Methods

### Study design and procedures

TRIBE^12^ and TRIBE2^15^ are two phase III randomized, open-label, multicenter trials involving 1187 patients with initially unresectable and previously untreated mCRC. In the TRIBE study, 508 patients were randomized in a 1 : 1 ratio to receive FOLFIRI/bevacizumab or FOLFOXIRI/bevacizumab, whereas in the TRIBE2 trial, 679 patients were randomized in a 1 : 1 ratio to receive FOLFOX/bevacizumab followed by FOLFIRI/bevacizumab after PD or FOLFOXIRI/bevacizumab followed by the reintroduction of the same agents after PD. All treatments were administered up to 12 cycles in TRIBE and up to 8 cycles in TRIBE2, followed by 5-fluorouracil plus bevacizumab until PD, unacceptable adverse events, or consent withdrawal in both trials. All the patients had a baseline computed tomography scan in the screening window and repeated the computed tomography scan at the prespecified timepoints to assess treatment response. Positron emission tomography scan or bone scintigraphy was not mandatory and carried out according to investigators’ decision.

### Statistics

The χ^2^ test and two-tailed Fisher’s exact test was used, when appropriate, to compare clinical and biological features. PFS and OS were determined according to the Kaplan–Meier estimates method and survival curves were compared using the log-rank test. HRs and 95% CIs were estimated with a Cox proportional hazards model. Odds ratios (ORs) and relative CIs were estimated with a logistic regression model.

The association of bone involvement with PFS and OS was first assessed in univariate analyses. The same analyses were carried out to evaluate the association of other potentially prognostic clinical [Eastern Cooperative Oncology Group (ECOG) performance status (PS), age, sex previous adjuvant therapy, time to metastases, surgery on primary tumor, primary tumor site, treatment arm] and molecular (*RAS* and *BRAF* mutational status) variables with PFS and OS. Significantly prognostic variables (*P* < 0.10) were included in a multivariate Cox proportional hazards model.

All statistical tests were two-sided, and *P-*values ≤0.05 were deemed significant. Statistical analyses were carried out using SAS version 9.4 (SAS Institute, Inc., Cary, NC). The data cut-off for the present analysis was 31 July 2014 and 30 July 2019 for TRIBE and TRIBE2, respectively.

## Results

As shown in Table 1, 41/1187 (3.5%) patients included in the intention-to-treat group had bone metastases at the baseline assessment. No relevant differences among subgroups according to other baseline characteristics were evident. Overall, 14 patients experienced an SRE during the trial; among them, 6 were enrolled in the TRIBE trial (4 in the triplet arm and 2 in the doublet arm), and 8 in the TRIBE2 trial (5 in the triplet and 3 the doublet). Among the six patients reporting an SRE in the TRIBE trial, five had skeletal RT and one had surgery; in the TRIBE2 trial, all the SREs were related to radiotherapy.

**Table 1:** Patients’ baseline characteristics

Intention-to-treat population	Bone involvement at progression (*n* = 1146), *n* (%)		*P*-value
	Yes	No	
**Treatment arm**			0.739
**Doublets + Bev**	19 (47)	555 (50)	
**FOLFOXIRI + Bev**	21 (53)	551 (50)	
**Sex**			0.081
**Male**	18 (45)	651 (59)	
**Female**	22 (55)	455 (41)	
**ECOG PS**			0.309
**0**	33 (83)	972 (88)	
**1-2**	7 (17)	134 (12)	
**Site of primary tumor**			0.902
**Right**	14 (35)	378 (34)	
**Left and rectum**	25 (63)	704 (64)	
**Unknown**	1 (2)	24 (2)	
**Resected primary tumor**			0.058
**Yes**	29 (73)	635 (57)	
**No**	11 (27)	471 (43)	
**Adjuvant treatment**			0.744
**Yes**	3 (7)	73 (7)	
**No**	37 (93)	1033 (93)	
**Age group (years)**			0.186
**<70**	37 (92)	929 (84)	
**≥70**	3 (8)	177 (16)	
**Time to metastases**			0.171
**Synchronous**	31 (77)	944 (85)	
**Metachronous**	9 (23)	162 (15)	
**Mutational status**			0.390
**Wild type**	6 (15)	222 (20)	
**RAS mut**	28 (70)	623 (56)	
**BRAF mut**	2 (5)	86 (8)	
**Unknown**	4 (10)	175 (16)	

BEV, bevacizumab; ECOG PS, Eastern Cooperative Oncology Group performance status.

**Figure 1:**
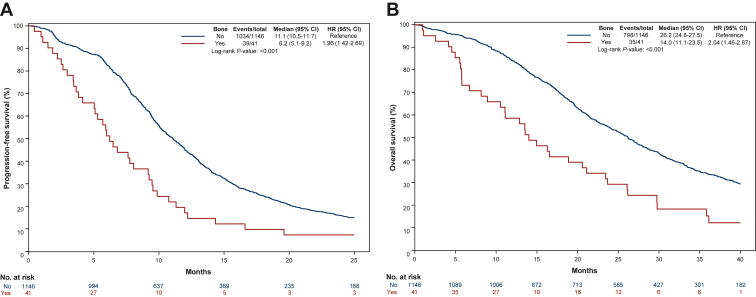
Kaplan–Meier curves showing differences in (A) progression-free survival and (B) overall survival in patients with and without bone metastases at baseline. CI, confidence interval; HR, hazard ratio.

In the whole population, patients with bone involvement at baseline reported shorter PFS (median PFS: 6.2 versus 11.1 months, HR 1.96, 95% CI 1.42-2.69; *P* < 0.001; Figure 1A) and OS (median OS: 14.0 versus 26.2 months, HR 2.04, 95% CI 1.46-2.87; *P* < 0.001; Figure 1B). The multivariable models confirmed the association of bone metastases with worse PFS (HR 2.37, 95% CI 1.67-3.37; *P* < 0.001; Table 2) and OS (HR 2.24, 95% CI 1.54-3.26; *P* < 0.001; Table 3)*.* As previously described, the other baseline factors associated with OS in the multivariate model were ECOG PS, previous resection of the primary tumor, sidedness, and mutational status.^16^^,^^17^^,^^14^ No difference was shown in terms of ORR (46% versus 58%; OR 0.63, 95% CI 0.33-1.17; *P* = 0.14) in patients with and without bone metastases at baseline.

**Table 2:** Univariate and multivariate analyses for progression-free survival

Characteristics	Values (*n* = 1187), *n* (%)	Progression-free survival			
		Univariate analysis		Multivariate analysis	
		HR (95% CI)	*P*-value	HR (95% CI)	*P*-value
**Bone**					
No	1146 (97)	1	**<0.001**	1	**<0.001**
Yes	41 (3)	1.96 (1.42-2.70)		2.37 (1.67-3.37)	
**Arm**					
Doublet + Bev	596 (50)	1	**<0.001**	1	**<0.001**
FOLFOXIRI + Bev	591 (50)	0.76 (0.68-0.86)		0.76 (0.66-0.87)	
**Age, years**					
<70	1005 (85)	1	0.436	—	—
≥70	182 (15)	1.07 (0.91-1.26)		—	
**ECOG PS**					
0	1038 (87)	1	**<0.001**	1	**<0.001**
1-2	149 (13)	1.78 (1.49-2.12)		1.72 (1.42-2.09)	
**Sex**					
Female	494 (42)	1	0.066	1	**0.027**
Male	693 (58)	1.12 (0.99-1.27)		1.17 (1.02-1.34)	
**Previous adjuvant therapy**					
No	1108 (93)	1	**0.004**	1	0.512
Yes	79 (7)	0.71 (0.56-0.91)		0.89 (0.62-1.27)	
**Time to metastases**					
Metachronous	179 (15)	1	**0.002**	1	0.516
Synchronous	1008 (85)	1.31 (1.10-1.55)		1.09 (0.85-1.39)	
**Resected primary tumor**					
No	499 (42)	1	**<0.001**	1	**0.008**
Yes	688 (58)	0.75 (0.66-0.84)		0.82 (0.71-0.95)	
**Site of primary tumor**					
Right	408 (34)	1	0.100	1	0.645
Left and rectum	751 (64)	0.90 (0.79-1.02)		0.97 (0.84-1.12)	
Unknown	28 (2)	—		—	
**Mutational status**					
All WT	237 (20)	1		1	
RAS mut	672 (57)	1.20 (1.03-1.40)	**0.022**	1.22 (1.04-1.43)	**0.016**
BRAF mut	94 (8)	1.71 (1.33-2.20)	**<0.001**	1.75 (1.34-2.29)	**<0.001**
Unknown	184 (15)	—		—	

Bold values are significant *P* values.

**Table 3:** Univariate and multivariate analyses for overall survival

Characteristics	Values (*n* = 1187), *n* (%)	Overall survival			
		Univariate analysis		Multivariate analysis	
		HR (95% CI)	*P*-value	HR (95% CI)	*P*-value
**Bone**					
No	1146 (97)	1	**<0.001**	1	**<0.001**
Yes	41 (3)	2.04 (1.46-2.87)		2.24 (1.54-3.26)	
**Arm**					
Doublet + Bev	596 (50)	1	**0.007**	1	0.037^a^
FOLFOXIRI + Bev	591 (50)	0.83 (0.72-0.95)		0.85 (0.73-0.99)	
**Age, years**					
<70	1005 (85)	1	0.107^a^	—	—
≥70	182 (15)	1.16 (0.97-1.40)		—	
**ECOG PS**					
0	1038 (87)	1	**<0.001**	1	**<0.001**
1-2	149 (13)	2.24 (1.86-2.70)		2.14 (1.75-2.62)	
**Sex**					
Female	494 (42)	1	0.180^a^	—	—
Male	693 (58)	1.10 (0.96-1.26)		—	
**Previous adjuvant therapy**					
No	1108 (93)	1	**<0.001**	1	0.357
Yes	79 (7)	0.57 (0.42-0.77)		0.82 (0.53-1.26)	
**Time to metastases**					
Metachronous	179 (15)	1	**<0.001**	1	0.312
Synchronous	1008 (85)	1.53 (1.24-1.87)		1.16 (0.87-1.55)	
**Resected primary tumor**					
No	499 (42)	1	**<0.001**	1	**0.002**
Yes	688 (58)	0.70 (0.61-0.80)		0.77 (0.66-0.91)	
**Site of primary tumor**					
Right	408 (34)	1	**<0.001**	1	**0.002**
Left and rectum	751 (64)	0.74 (0.64-0.85)		0.83 (0.71-0.98)	
Unknown	28 (2)	—		—	
**Mutational status**					
All WT	237 (20)	1		1	
RAS mut	672 (57)	1.65 (1.37-2.00)	<0.001^a^	1.64 (1.35-2.00)	<0.001^a^
BRAF mut	94 (8)	2.96 (2.24-3.92)	<0.001^a^	2.85 (2.12-3.83)	<0.001^a^
Unknown	184 (15)	—		—	

Bold values are significant *P* values.

a Non-significant *P* values.

**Figure 2:**
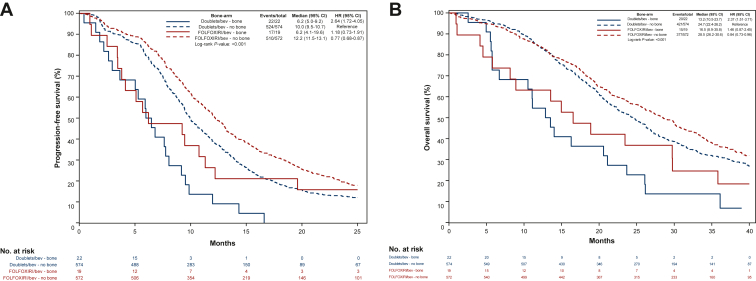
Kaplan–Meier curves showing differences in in (A) progression-free survival and (B) overall survival in patients with and without bone metastases according to treatment arm. BEV, bevacizumab.

With regard to treatment efficacy, no interaction effect was found between bone involvement and treatment arm for both PFS (*P* = 0.094; Figure 2A) and OS (*P* = 0.38; Figure 2B).

After the first progression, 77/1070 (7%) alive patients had bone metastases at the time of progression and reported shorter survival after progression (10.4 versus 13.2 months; HR 1.48 95% CI 1.15-1.91; *P* = 0.002) compared with patients with no bone involvement (Supplementary Figure S1A, available at https://doi.org/10.1016/j.esmoop.2022.100606). A trend for shorter survival was reported among patients with bone metastases at baseline (*n* = 37) as compared with those with new bone lesions at the time of progression (*n* = 40; 7.5 versus 11 months, HR 1.50, 95% CI 0.92-2.45; *P* = 0.10; Supplementary Figure S1B, available at https://doi.org/10.1016/j.esmoop.2022.100606).

We then carried out a subgroup analysis on patients who developed bone metastases at first radiological progression, excluding those with bone metastases at baseline. Overall, 40 (3%) patients reported new bone lesions at the time of progression, whereas 1106 (97%) never experienced skeletal involvement. No difference was present in terms of baseline characteristics (Table 1).

The two groups did not differ in terms of ORR (60% versus 57%; OR 1.088, 95% CI 0.571-2.070; *P* = 0.798). When comparing median PFS, patients with bone progression at first radiological progression reported a trend toward shorter PFS (9.0 versus 11.1 months, HR 1.35, 95% CI 0.98-1.85; *P* = 0.06) compared with those who progressed in other sites, even if the data are not statistically significant.

## Discussion

The prognostic and predictive impact of bone metastases in CRC has been poorly investigated. Data from large clinical trials are currently lacking and most of the evidence comes from retrospective series.^9^^,^^18^ According to available data, skeletal involvement is associated with worse survival,^6^ and little is known about its predictive implications. Based on the aforesaid considerations, we carried out a pooled analysis of the TRIBE^12^ and TRIBE2^15^ trials, to assess the prognostic and predictive significance of bone metastases in patients receiving doublet or triplet chemotherapy plus bevacizumab.

In our study population, patients with bone lesions reported significantly worse PFS and OS compared with patients without bone metastases. The independent prognostic role of baseline bone metastases was confirmed in the multivariate models for both OS and PFS, along with other well-established prognostic factors.^16^^,^^17^ Moreover, patients who developed skeletal metastases at first radiological progression (PFS1) had a worse OS compared with patients who experienced progression in other sites. When analyzing separately those who experienced new bone lesions at the time of progression (excluding those with baseline bone metastases), they were found not to be different in terms of baseline characteristics and ORR from the overall population without skeletal metastases.

However, this subgroup of patients showed a trend toward a shorter PFS1 compared with the counterpart free from bone metastases; the absence of statistical significance could be explained by the small sample size. These data, despite being nonsignificant, confirmed the more aggressive behavior of bone-involving CRCs. Anyway, the comparable ORR of both groups, even in the presence of skeletal progression, reflects the preserved activity of chemotherapy backbones even in this prognostic unfavorable subgroup.

In the overall population, patients with skeletal involvement at baseline had a nonsignificant trend toward worse survival compared with those who developed bone metastases at first radiological progression. This could be explained by the higher disease burden usually associated with synchronous bone lesions. In our analyses, skeletal involvement had no predictive role. In fact, no interaction effect was evident between the presence of bone metastases and the chemotherapy intensity in terms of both PFS and OS. The survival advantage of FOLFOXIRI plus bevacizumab observed in the overall population of the TRIBE and TRIBE2 trials was retained in the limited subgroup of patients with bone lesions.

Our results, from a large cohort of patients enrolled in two phase III studies, provide robust information on prognostic and predictive factors for patients with mCRC; however, some limitations apply to our study. Because of the retrospective nature of this analysis, no data were collected regarding the use of antiresorptive agents or other bone-directed therapies. Furthermore, the low incidence of SREs does not allow comparisons between the treatment arms.

In conclusion, this pooled analysis confirmed the negative prognostic value of bone metastases in CRC. Triplet regimen maintained a survival advantage even in the presence of bone metastases and this indicates that the association of bevacizumab plus FOLFOXIRI is a valid therapeutic option in this subset of patients at poor prognosis. However, specific prospective studies are warranted to confirm this finding and to identify, in this particular group, those who could benefit the most from more intensive treatment. Therefore more efforts are needed to clearly understand the biology and the clinical course of bone metastases in CRC, to provide the best treatment to this difficult subset of patients.

## Supplementary data

Supplementary Figure S1 Kaplan-Meier curves showing differences in overall survival (OS) in patients with and without bone involvement at PD (A) and in patients with bone involvement at baseline and bone involvement at PD (B).
