# Supplementary figures and images for: Bone metastases are associated with worse prognosis in patients affected by metastatic colorectal cancer treated with doublet or triplet chemotherapy plus bevacizumab: a subanalysis of the TRIBE and TRIBE2 trials
Source: ESMO Open. 2022 Oct 31;7(6):100606. doi: 10.1016/j.esmoop.2022.100606 (PMC9808439; doi:10.1016/j.esmoop.2022.100606)

A

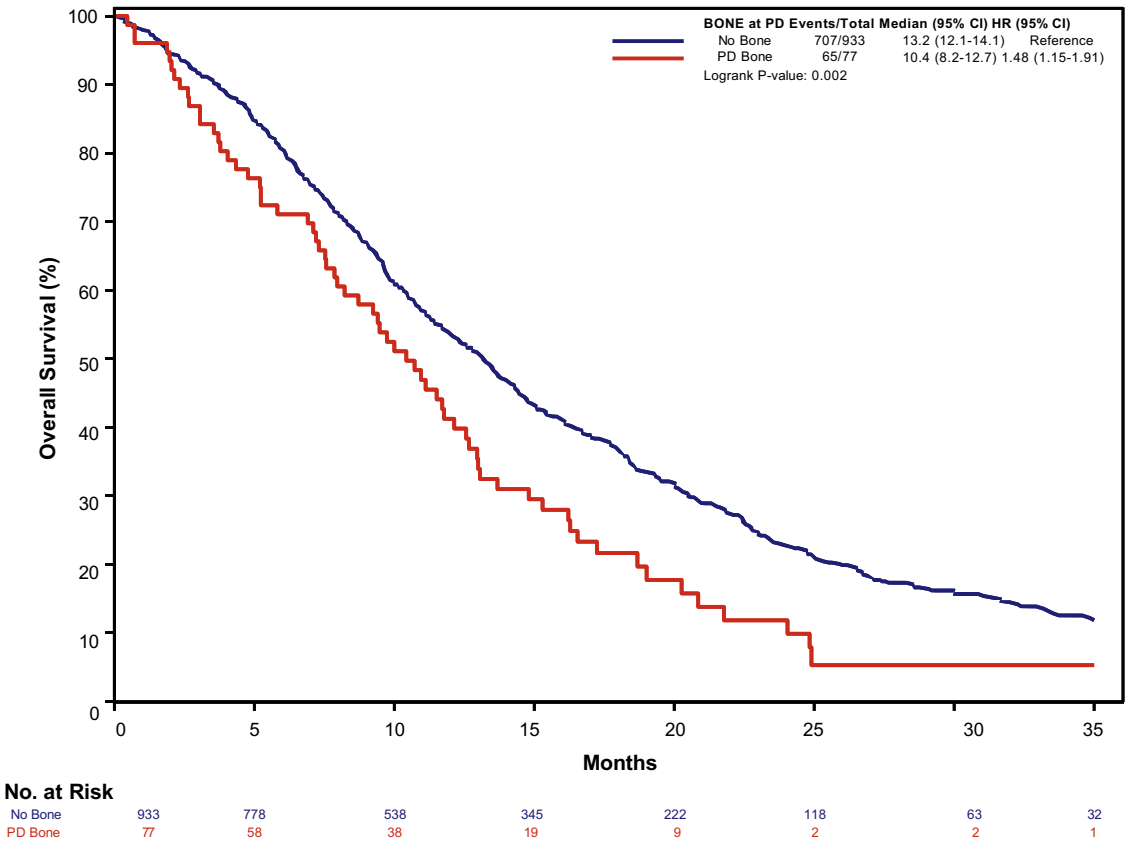

B

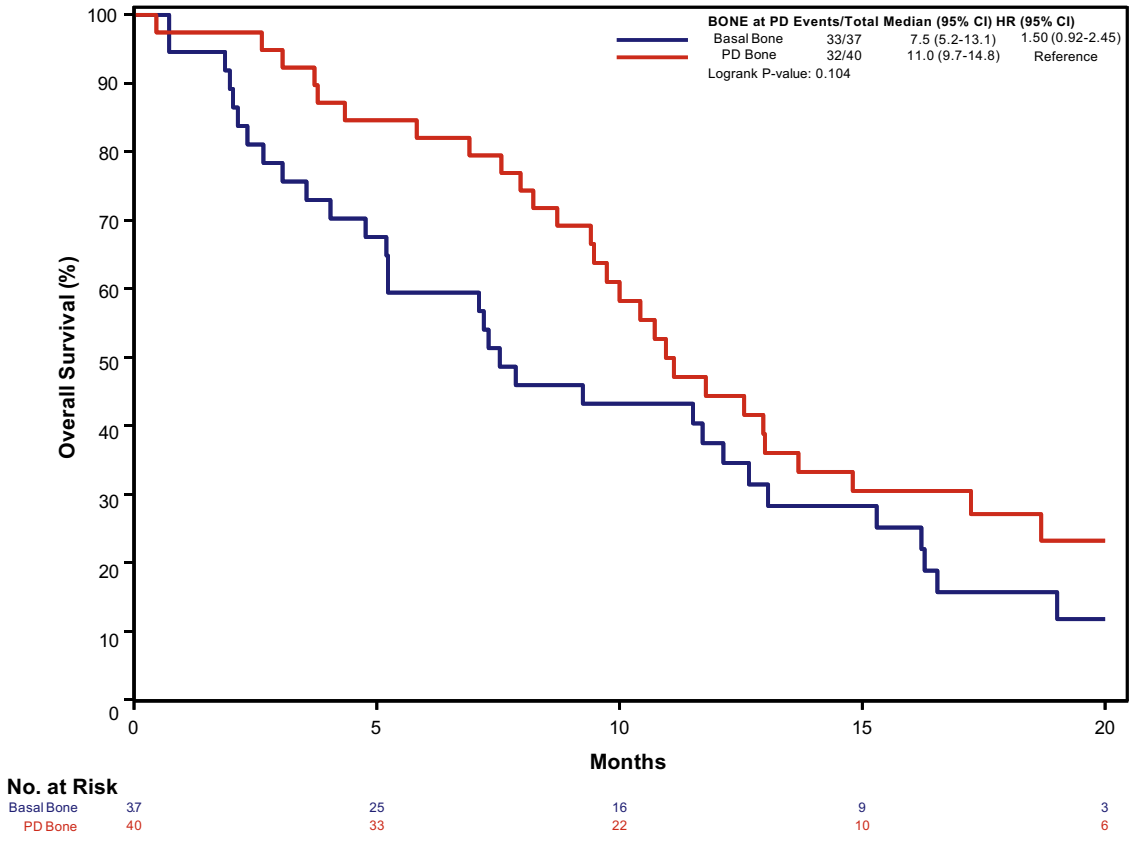

Supplement: Supplementary Figure S1 — Kaplan-Meier curves showing differences in overall survival (OS) in patients with and without bone involvement at PD (A) and in patients with bone involvement at baseline and bone involvement at PD (B). [file mmc1.pdf]
